# The Alzheimer’s disease risk factors apolipoprotein E and TREM2 are linked in a receptor signaling pathway

**DOI:** 10.1186/s12974-017-0835-4

**Published:** 2017-03-21

**Authors:** Charlotte Jendresen, Vibeke Årskog, Michael R. Daws, Lars N. G. Nilsson

**Affiliations:** 1 Department of Pharmacology, Institute of Clinical Medicine, University of Oslo and Oslo University Hospital, Postboks 1057 Blindern, 0316 Oslo, Norway; 2 Division of Anatomy, Institute of Basic Medical Sciences, University of Oslo, Oslo, Norway

**Keywords:** Alzheimer’s disease, Apolipoprotein E (ApoE), Triggering receptor expressed on myeloid cells 2 (TREM2), Reporter cells, Neuroinflammation

## Abstract

**Background:**

Triggering receptor expressed on myeloid cells 2 (*TREM2*) and apolipoprotein E (*APOE*) are genetically linked to Alzheimer’s disease. Here, we investigated whether human ApoE mediates signal transduction through human and murine TREM2 and sought to identify a TREM2-binding domain in human ApoE.

**Methods:**

To investigate cell signaling through TREM2, a cell line was used which expressed an *NFAT*-inducible β-galactosidase reporter and human or murine TREM2, fused to CD8 transmembrane and CD3ζ intracellular signaling domains. ELISA-based binding assays were used to determine binding affinities of human ApoE isoforms to human TREM2 and to identify a TREM2-binding domain in ApoE.

**Results:**

ApoE was found to be an agonist to human TREM2 with EC_50_ in the low nM range, and to murine TREM2 with reduced potency. In the reporter cells, TREM2 expression was lower than in nontransgenic mouse brain. Human ApoE isoforms ε2, ε3, and ε4 bound to human TREM2 with *K*_d_ in the low nM range. The binding was displaced by an ApoE-mimetic peptide (amino acids 130–149).

**Conclusions:**

An ApoE-mediated dose-dependent signal transduction through TREM2 in reporter cells was demonstrated, and a TREM2-binding region in ApoE was identified. The relevance of an ApoE-TREM2 receptor signaling pathway to Alzheimer’s disease is discussed.

**Electronic supplementary material:**

The online version of this article (doi:10.1186/s12974-017-0835-4) contains supplementary material, which is available to authorized users.

## Background

Triggering receptor expressed on myeloid cells 2 (TREM2) is part of the immunoglobulin-lectin-like receptor superfamily, and in the brain, TREM2 is expressed mainly in microglial cells. TREM2 couples to DNAX-activating protein of 12 kDa (DAP12) and activates its downstream targets through the phosphorylation of the immunoreceptor tyrosine-based activation motif (ITAM) of DAP12. TREM2 function has been related to phagocytosis, cell growth, regulation of actin cytoskeleton, migration towards chemokines, and cytokine release (reviewed in [1, 2]). The inheritance of a mutant variant of the *TREM2* gene, *R47H*, confers a markedly increased risk for developing late-onset [3, 4] and early-onset [5] Alzheimer’s disease (AD). TREM2 expression has been found in the close vicinity of amyloid plaques in AD brain and transgenic mouse models, particularly in microglia and infiltrating macrophages surrounding plaques [6, 7]. Moreover, loss-of-function mutations in *TREM2* are linked to an increased risk of developing Nasu-Hakola disease, frontotemporal dementia, Parkinson’s disease, and sporadic amyotrophic lateral sclerosis [8–13]. This suggests a significant role for TREM2 in neurodegenerative diseases with phenotypes being dependent upon the location of the mutation and the severity of protein dysfunction [14]. Since *TREM2* variants seem broadly involved in neurodegeneration, there is an urgent need to further investigate the functions of TREM2 in the brain and to find ligands involved in TREM2-mediated signaling and their role in AD pathogenesis.

Another strong risk factor for developing late-onset AD is the *ε4* allele of the apolipoprotein E gene (*APOE*), which is far more prevalent than the *R47H-TREM2* risk factor [3]. The three major isoforms of ApoE in human are the ApoE ε2 (cys112, cys158), ApoE ε3 (cys112, arg158), and ApoE ε4 (arg112, arg158). The most common isoform is ApoE ε3, while ApoE ε2 isoform is rare. The *APOE4* allele increases the risk of AD by three- to fourfold [15, 16], while *APOE2* is protective [17]. The disease risk is gene dose-dependent for both alleles [15–17]. In the brain, astrocytes are the main source of ApoE, and ApoE is the major regulator of lipid metabolism, transporting cholesterol and phospholipids between cells (reviewed in [18]). ApoE is intimately associated with fibrils of amyloid-β (Aβ) in amyloid deposits in AD brain and transgenic animal models of AD [16, 19, 20]. Early studies reported both inhibition and augmentation of Aβ fibril formation when mixing ApoE and Aβ in vitro [21–25]. Some studies reported that the ApoE ε4 isoform bound faster to Aβ and increased aggregation more readily than the ApoE ε2 or ε3 isoforms [23, 25–27]. The interpretation of such studies in relation to the brain is complicated by the complex nature of Aβ (mixture of different lengths and aggregation states) and ApoE (isoform and lipidation state), which is difficult to mimic in vitro. A link between human ApoE and amyloid plaque accumulation in amyloid-β precursor protein (AβPP) transgenic mouse brain has been consistently found [28–30]. Since mice have an ApoE isoform distinct from the human isoforms, these studies have mostly been done on AβPP transgenic mice crossed with *Apoe* knockout mice (*Apoe* ^−/−^) instead expressing human *APOE*. Such studies have shown that murine ApoE augments Aβ deposition more than either of the human ApoE isoforms and that the order of fibrillogenic effect on Aβ of the human ApoE isoforms is ApoE ε4 > ApoE ε3 > ApoE ε2 [28, 31, 32]. These results are consistent with the relative risk associated with the ApoE variants for developing AD. The effect of ApoE on Aβ aggregation also depends on the human ApoE concentration [30], as also reported for murine ApoE in the first AβPP/ApoE cross-breeding study [33].

All three main human ApoE isoforms were recently found to bind to human TREM2 in vitro [34, 35]. In a recent paper, these findings were partly opposed, as binding could not be detected between human TREM2 and nonlipidated human ApoE using protein microarray, while lipidated human ApoE bound TREM2 when bio-layer interferometry was used [36]. The reported experiments were all done in pure in vitro (cell-free) settings [34–36]. It remains unclear whether ApoE binds to TREM2 when it resides on cell surfaces, and whether ApoE serves as a TREM2 agonist. Experimental proof of such an interaction would effectively link the two major genetic risk factors for AD in a signaling pathway.

In this study, we used a cell reporter assay to provide the first evidence of human ApoE-mediated intracellular signaling through human TREM2, which is of great importance for understanding of the interaction between ApoE and TREM2 in the AD pathogenesis. Possible interactions between human ApoE and murine TREM2 have never been examined despite multiple studies using transgenic AβPP mice expressing human ApoE instead of murine ApoE. We report that human ApoE signals through murine TREM2, albeit with a reduced efficacy. The *K*_d_ has previously only been determined for the ApoE ε3 isoform using dot blotting [34], a semi-quantitative method. We developed a sensitive ELISA-based binding assay to determine the affinity of human TREM2 binding to the three major human ApoE isoforms and, importantly, identified a TREM2-binding region in human ApoE, which has never been reported before.

## Methods

### Reagents

Transparent Maxisorp 96-well plates (#442404), Roswell Park Memorial Institute 1640 medium (RPMI; #61870-044), Zeocin (#R250-01), ionomycin (#I-24222), 2-mercaptoethanol (#31350-010), ethylenediaminetetraacetic acid (EDTA; #15575-020), trypan blue (#T10282), as well as secondary horseradish peroxidase (HRP)-conjugated rabbit anti-goat (#31402) and goat anti-mouse (#31430) antibodies were from Thermo Fischer Scientific (Waltham, MA, USA). Phorbol myristate acetate (PMA; #P1585), chlorophenol-red β-D-galactopyranoside (CPRG; #59767), magnesium chloride hexahydrate (#M9272), potassium chloride (#P5405), sodium phosphate dibasic dehydrate (#30412), sodium chloride (#71376), phosphate-buffered saline (PBS, pH 7.4; #P4417), sodium dodecyl sulfate (SDS; #L4390), Roche complete protease inhibitors (#04-693-116-001), fetal bovine serum (FBS; #F7524), and bovine serum albumin (BSA; #A7030) were from Sigma-Aldrich (St. Louis, MO, USA). Monoclonal antibodies against β-actin (clone AC-15) and FLAG-peptide sequence DYKDDDDK (M2; #F1804) were also from Sigma-Aldrich. Molecular biology grade 2-mercaptoethanol (#A1108) was from AppliChem (Darmstadt, Germany). Electran 1,4-dithiothreithol (Cleland’s reagent, DTT; #443853B) and KPL LumiGLO kit (#54-71-00) were from VWR (Radnor, PA, USA). 7.5 and 12% TGX polyacrylamide gels (Mini-protean; #4561026 and #4561043, respectively) were from Bio-Rad (Hercules, CA, USA). Saponin (#558255) and polyclonal goat anti-ApoE antibody (#178479) were from Merck Millipore (Darmstadt, Germany). Polyclonal goat anti-TREM2 antibody (#ab95470), preadsorbed unconjugated polyclonal rabbit anti-rat antibody (#ab102248) and secondary HRP-conjugated donkey anti-goat antibody (#ab7125) were from Abcam (Cambridge, UK). Recombinant human ApoE ε2, ApoE ε3, and ApoE ε4 (#4760, #4696, and #4699, respectively) were bought from BioVision (Milpitas, CA, USA). Recombinant human TREM2 (#11084-H08H) and mouse TREM2 (#50149-M08H) were bought from Sino Biological Inc. (Beijing, China). An ApoE-mimetic peptide corresponding to amino acids 130–149 (acetyl-TEELRVRLASHLRKLRKRLL-amide) and a scrambled peptide serving as negative control (acetyl-LREKKLRVSALRTHRLELRL-amide) were from GenScript (Piscataway, NJ, USA). K-blue aqueous substrate (TMB; #331177) was bought from ANL-Produkter (Älvsjö, Sweden). Monoclonal rat antibodies were made towards TREM2 (clone 150; [37]) and T cell immunoglobulin domain and mucin domain 2 (TIM2) (clone N3-4; [38]).

### Reporter cell lines

**Fig. 1:**
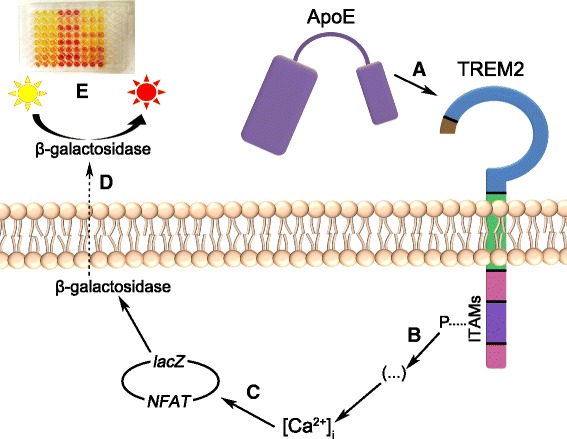
Signal transduction mechanism and detection principle of the BWZ reporter assays. In the BWZ cells, TREM2 (shown in *blue*) is expressed in a single chimeric protein containing CD8 transmembrane domain (shown in *green*) and a CD3ζ domain (shown in *purple* and *pink*), with an extracellular FLAG-tag (shown in *brown*). When a TREM2 ligand, here ApoE, binds to TREM2 (**a**), the CD3ζ ITAM motifs (shown in *purple*) are phosphorylated (**b**), which leads to an intracellular signaling cascade resulting in an increased intracellular Ca^2+^ concentration. This induces the NFAT promoter, so that β-galactosidase is expressed from the *lacZ* gene (**c**). β-galactosidase is released from the cells using a lysis buffer containing saponin (**d**). The *yellow* β-galactosidase substrate, CPRG, is also present in the lysis buffer, and when cleaved by β-galactosidase, it forms a *red* product which is measured by absorbance (**e**)

To investigate stimulation of TREM2 by ApoE, we used a reporter cell line based on T lymphocytes in which the α- and β-beta chains of the endogenous T cell receptor had been knocked out, and the cells expressed *lacZ* under a nuclear factor of activated T cells (*NFAT*) promotor in an overexpression system [39, 40]. The cells were used to generate the reporter cells that also express human or murine TREM2. Here, TREM2 was linked in a single transmembrane fusion construct to a chimeric antigen receptor with the leader sequence and transmembrane CD8 domain fused with the intracellular CD3ζ. When an agonist binds to TREM2, the ITAMs of CD3ζ are phosphorylated, starting an intracellular signaling cascade through zeta-chain-associated protein kinase 70 (ZAP-70) thereby increasing the intracellular Ca^2+^ concentration. This resembles the physiological signaling of TREM2 through DAP12, in which ITAM-phosphorylation also recruits ZAP-70 [41]. The *NFAT* promotor is activated in response to the increased intracellular Ca^2+^ concentration [39, 40] such that β-galactosidase is expressed. Since β-galactosidase is expressed as a result of TREM2 signal transduction and *NFAT* promoter activity, we can determine the level of TREM2-stimulation by measuring the product generated by a colorimetric β-galactosidase substrate, CPRG (Fig. 1).

All cells were grown in RPMI supplemented with 1% penicillin + streptomycin, 5% FBS, 50 μM EDTA, and 50 μM 2-mercaptoethanol (termed cRPMI) in a cell incubator with 37 °C and 5% CO_2_. The BWZ.36 cells are based on an immortalized T lymphocyte cell line [39, 40] and constitutively express 3x*NFAT/LacZ*. Naïve BWZ.36 cells were transfected by electroporation with human *TREM2-CD8-CD3ζ* pcDNA4 (BWZ-hTREM2), murine *Trem2-CD8-CD3ζ* pcDNA4 (BWZ-mTREM2), or TIM2-CD8-CD3ζ pcDNA4 (BWZ-TIM2), all with a FLAG-tag sequence incorporated as described elsewhere [37, 38]. TIM2 shows partial homology to the Ig domain of TREM2 [38]. Successfully transfected cells were selected with Zeocin (0.75 mg/mL).

### Reporter assays

For testing stimulation of human and murine TREM2, we used a modified version of a reporter cell assay originally described by Sanderson and Shastri [40]. Cells used in reporter assays were counted using 0.2% trypan blue and Invitrogen Countess automated cell counter (Thermo Fischer Scientific) and used only if the viability was above 90%.

Transparent Maxisorp 96-well plates were used for the reporter assays. All coatings were done for 1 h at 37 °C in sterile, special PBS with reduced ionic strength (S-PBS; pH 7.4): 10 mM Na_2_HPO_4_, 2 mM KH_2_PO_4_, 13.7 mM NaCl, and 0.27 mM KCl. The wells of the plate were coated either with 2.5 μg/mL preadsorbed, unconjugated polyclonal rabbit anti-rat antibody, recombinant human ApoE ε2, ApoE ε3, or ApoE ε4 (3–300 nM diluted in S-PBS), or S-PBS alone. Between coating and adding BWZ cells to the plate (2.4 × 10^5^ cells in cRPMI per well), the wells were carefully washed once with sterile S-PBS. All BWZ cells were mixed with different stimuli together with 3.3 nM PMA as a costimulating agent and incubated for 4 h in a cell chamber with 37 °C and 5% CO_2_. The stimuli used were 3.7 or 5.2 nM monoclonal rat anti-TIM2 antibody [38] or 3.3 nM monoclonal rat anti-TREM2 antibody (“ab150”). The Ab150 antibody, also called clone 150.1, was produced at the same time as clone 78.18 referenced in [42], although ab150 (clone 150.1) is subclass IgG2a, while 78.18 is IgG1 [43]. Clone 150.1 detects both human and murine TREM2 in flow cytometry (unpublished data) and stimulates both human and murine TREM2 in our reporter assays. Thus, ab150 was used as a positive control for TREM2 receptor signaling, while the anti-TIM2 antibody was used as a positive control for TIM2 receptor signaling. After incubation with the stimuli, the cells were carefully washed once with Hi-S-PBS (high phosphate content and low saline content: 60.7 mM Na_2_HPO_4_, 13.2 mM KH_2_PO_4_, 0.27 mM KCl, and 13.7 mM NaCl). Finally, freshly prepared lysis buffer with CPRG substrate was added to the wells: 3 mM CPRG in Hi-S-PBS with 5 mM DTT, 20 mM MgCl_2_, and 0.2% (*w*/*v*) saponin. Volumes of added cells, coating solutions, washing solutions, and lysis buffers were all 200 μL/well.

The absorbance was monitored from once the enzymatic reaction had started and onwards for 4 h at 570 nm (specific signal) and 700 nm (background) with a SpectraMax 190 plate reader (Molecular Devices). The plate was tightly sealed and placed gently shaking at 37 °C between measurements. The absorbance at λ_700 nm_ background was subtracted from the specific signal at λ_570 nm_ when processing the data. All results presented in this paper are from 4 h after adding the CPRG-containing lysis buffer to the stimulated cells. All measurements were made in triplicate.

### Protein extraction and western blot

BWZ reporter cells, 14-month-old nontransgenic C57BL/6 J mouse brain, and 6-month-old *Trem2*-knockout mouse brain were extracted with a Potter-Elvehjem homogenizer (#432-5015; VWR) by manual homogenization on ice with two times of ten strokes in ice-cooled 1% (*w*/*v*) SDS in 20 mM Tris-HCl (pH 7.5), 137 mM NaCl, and 4 mM EDTA with complete protease inhibitors. The resulting homogenate was centrifuged at 100,000*g* for 1 h at +4 °C in a Beckman Optima LE-80 K ultracentrifuge with a Sw-60Ti rotor. After centrifugation, the supernatant was stored at −80 °C until use. SDS-protein extracts (30–50 μg total protein) were mixed with Laemmli buffer containing β-mercaptoethanol (1% *v*/*v*) and bromophenol blue (62.5 mg/L), heat-denatured at 75 °C for 5 min, and separated by electrophoresis at 160 V. Proteins were transferred to nitrocellulose membranes with Trans-Blot semi-dry transfer system at 20 V for 30 min. The filters were blocked for 1 h at 37 °C with 5% (*w*/*v*) non-dry milk powder in a Tris-buffered saline (TBS) buffer containing 0.1% Tween-20 before being incubated with primary antibodies recognizing either murine TREM2 (0.1 μg/mL) or FLAG-tag M2-epitope (1 μg/mL) overnight at 4 °C. The membranes were then incubated 30 min at room temperature (RT) with secondary rabbit anti-goat (0.08 μg/mL) or goat anti-mouse (0.08 μg/mL) HRP-conjugated antibodies. The immunoreactive bands were visualized with enhanced chemiluminescence substrate using a LumiGLO kit. After stripping of the membranes, they were incubated for 30 min at RT with an antibody against β-actin (1:25000 dilution), followed by 30 min incubation at RT with secondary goat anti-mouse (0.08 μg/mL) HRP-conjugated antibody and development with LumiGLO. Optical densities of the immunoreactive bands were quantified with Image Pro Plus (Media Cybernetics, Rockville, MD, USA). Western blots were performed two to three times, and representative images are shown.

### In vitro binding assay

Transparent Maxisorp 96-well plates were coated with recombinant human TREM2 (40 nM) on a shaking platform for 1 h at 37 °C in sterile PBS (pH 7.4). The plates were blocked with 1% (*w*/*v*) BSA (fatty acid free) in PBS for 1 h at 37 °C before being incubated with recombinant human ApoE (ε2, ε3, or ε4) at various concentrations for 1 h at RT. The plates were then incubated for 1 h at RT with 12 ng/mL polyclonal goat anti-ApoE antibody followed by 1 h at RT with 20 ng/mL donkey anti-goat HRP-conjugated antibody. Finally, the assay was developed with K-blue aqueous substrate (TMB) for 15 min at RT, and the reaction was stopped with 1 M H_2_SO_4_. Absorbance was measured at 450 nm with a SpectraMax 190 plate reader (Molecular Devices). The plates were washed between each step with PBS with 0.05% (*w*/*v*) Tween-20.

### In vitro competition assay

Transparent Maxisorp 96-well plates were coated with recombinant human TREM2 (40 nM in sterile PBS) shaking for 90 min at 37 °C. The plates were blocked 1 h on a shaking platform at 37 °C with 1% (*w*/*v*) BSA (fatty acid-free) in PBS before being incubated 1 h at RT on a shaking platform with 6 nM recombinant human ApoE (ε2, ε3, or ε4) and various concentrations of ApoE-mim149 or scrambled peptide (0–6 μM). For detection of ApoE bound to TREM2, a polyclonal goat anti-ApoE antibody (18 ng/mL) was used that only detects full-length ApoE, but not ApoE-mim149 or scrambled peptide (Additional file 1: Figure S1) and incubated 1 h on shaker at RT followed by incubation with a donkey anti-goat HRP-conjugated antibody (33 ng/mL) for 30 min on shaker at RT. The assay was developed with K-blue aqueous substrate (TMB) for 30 min at RT, and the reaction was stopped with 1 M H_2_SO_4_. Absorbance was measured at 450 nm with a SpectraMax 190 plate reader (Molecular Devices).

### Statistics

All experiments were performed three times with triplicates unless otherwise stated. GraphPad Prism vs. 5.04 (San Diego, CA, USA) was used for graphs and statistical analyses. For analyzing dose-response and binding curves, data from at least three independent experiments were pooled and normalized. Normalization of all data was done using the formula *Y* = ((*X*−*L*)/(*K*−*L*))* 100% (in which *Y* represents the normalized data point, *X* represents the raw data point, *L* represents the mean of the 0% data point, which was the lowest concentration of ApoE used, and *K* represents the mean of the 100% data point with highest signals). *K*_d_, EC_50_, and Hill coefficients were calculated using the “log (agonist) vs. normalized response (variable)” function and were compared using one-way ANOVA with Bonferroni post-test. For the competition assay between full-length ApoE and ApoE-mim149, the *K*_i_ values were found by using the “one site-fit *K*_i_” function with 6 nM set as HotNM for all isoforms, and HotKdNM set as the respective ApoE isoform *K*_d_ values calculated in the binding assay. For comparing effects of adding ApoE-mim149 to full-length ApoE, data points were compared individually using an unpaired *t* test for each ApoE-mim149 concentration. All reported *p* values are two-tailed.

## Results

### ApoE ε3 stimulates human TREM2-, but not TIM2-transfected reporter cells

**Fig. 2:**
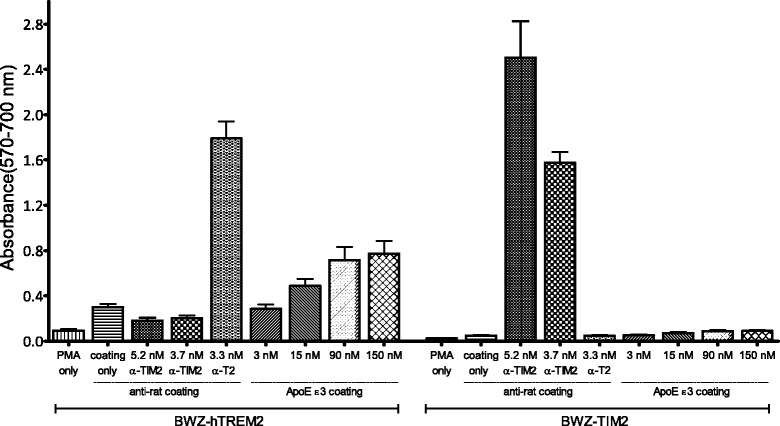
Dose-dependent ApoE ε3-induced signal transduction in BWZ reporter cells expressing human TREM2. A dose-dependent stimulation of signal transduction in BWZ-hTREM2 cells was observed when cells were incubated in wells coated with an increasing concentration of recombinant ApoE ε3. Stimulation of BWZ-hTREM2 cells was compared to stimulation of BWZ cells instead transfected with TIM2 (BWZ-TIM2). All cells were stimulated with PMA (3.3 nM). As a positive control of BWZ-hTREM2 stimulation, cells were incubated with a rat anti-TREM2 antibody (“α-T2”) in solution in wells coated with an anti-rat antibody. As a positive control for TIM2 stimulation, a rat anti-TIM2 antibody (“α-TIM2”) was used in conjunction with coating wells with anti-rat antibody similarly as for the BWZ-hTREM2 cells. Negative controls for both cell lines included cells stimulated only with PMA (“PMA only”), or cells growing in anti-rat coated wells (“coating only”). The columns depict mean ± SEM, and reflect data from three individual experiments with triplicates

We wanted to investigate whether stimulation with the most common human ApoE isoform, ApoE ε3, would induce human TREM2 signaling. For this, reporter cells were generated with *TREM2-CD8*-*CD3ζ* and *NFAT*/*lacZ* constructs. When an agonist binds to TREM2 in these cells, an intracellular signaling cascade is initiated through the ITAM motif of CD3ζ. As a result of extracellular TREM2-stimulation, β-galactosidase expression is induced by the *NFAT* promoter (Fig. 1). A colorimetric CPRG-assay was used to detect TREM2 signal transduction in the reporter cells. We compared stimulation of BWZ-hTREM2 cells to that of TIM2-transfected cells. TIM2 shows partial homology to the Ig domain of TREM2; the production of the cells is described in [38]. All cells were stimulated with PMA, and cells that were PMA-stimulated, but not incubated with any other stimuli, were used as negative controls. An anti-TREM2 antibody was used as positive control for stimulation of BWZ-hTREM2 receptor signaling. An anti-TIM2 antibody was used as positive control for stimulation of BWZ-TIM2 receptor signaling. We found that ApoE ε3 was an agonist for human TREM2 (Fig. 2) and demonstrated a dose-dependent signaling effect in BWZ-hTREM2 cells that markedly differed from BWZ-TIM2 cells. As might be noted in Fig. 2, the cells displayed a weak dose-dependent ApoE-mediated background (*p* < 0.05) that was independent of transfection with TREM2. This background was likely caused by ApoE acting on other receptors on the T lymphocytes, such as the low-density lipoprotein receptor (LDLR) [44–46], possibly resulting in a minor increase in the intracellular Ca^2+^ concentration. However, the potential background effects of stimulation of other receptors are small in comparison to stimulation of TREM2.

### ApoE ε2, ApoE ε3, and ApoE ε4 are all agonists for both human and murine TREM2

**Fig. 3:**
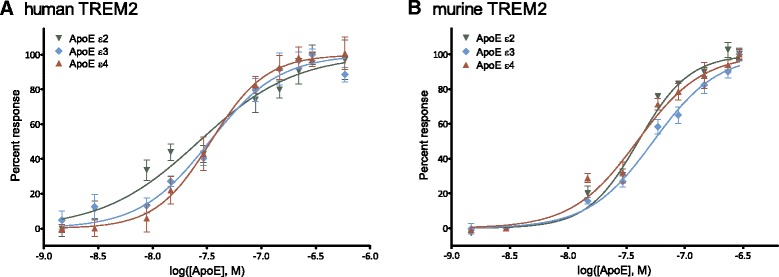
The three human ApoE isoforms serve as agonists for human and murine TREM2. Human ApoE ε2 (*inverted green triangle*), ε3 (*light blue diamond*), and ε4 (*red triangle*) all stimulated human (**a**) and murine (**b**) TREM2 receptor signal transduction in a dose-dependent manner. EC_50_ for human ApoE was 27, 33, and 34 nM for ApoE ε2, ε3, and ε4, respectively. EC_50_ for murine TREM2 was 40, 54, and 39 for ApoE ε2, ε3, and ε4, respectively. The points depict mean ± SEM of all responses measured in triplicates from five (human TREM2) and three (murine TREM2) independent experiments. Data from each ApoE isoform was normalized individually as described in the “Methods” section. The scale of the *x*-axis is logarithmic and shows concentration of human ApoE in M

Next, we examined if the other two human isoforms of ApoE, ε2 and ε4, were likewise agonists for human TREM2. For ApoE stimulation of human TREM2, EC_50_ was 27, 33, and 34 nM for ApoE ε2, ApoE ε3, and ApoE ε4, respectively (Fig. 3a; ε2 −logEC_50_ = 7.57 ± 0.06 M, ε3 −logEC_50_ = 7.48 ± 0.04 M, ε4 −logEC_50_ = 7.47 ± 0.05 M). There was no statistically significant difference in EC_50_ between the isoforms. We also performed the same experiments with murine TREM2 and determined EC_50_ for both human and murine TREM2. For murine TREM2, the EC_50_ was 40, 54, and 39 nM for ApoE ε2, ApoE ε3, and ApoE ε4, respectively (Fig. 3b; ε2 −logEC_50_ = 7.40 ± 0.02 M, ε3 −logEC_50_ = 7.27 ± 0.03 M, ε4 −logEC_50_ = 7.41 ± 0.04 M). The EC_50_ was significantly higher for ApoE ε3 than for ApoE ε2 and ε4 (*p* < 0.001 for both). There was no significant difference between ApoE ε2 and ApoE ε4 (*p* > 0.05). The dose-response curve for ApoE stimulation of human TREM2 showed Hill coefficients between 1 and 2 (ε2 1.0 ± 0.1, ε3 1.4 ± 0.2, ε4 1.7 ± 0.2 (Fig. 3a), with statistically significant difference between ApoE ε2 and ε4 (*p* < 0.001). The Hill coefficients for murine TREM2 were also between 1 and 2 (2.0 ± 0.1, 1.6 ± 0.1, and 1.6 ± 0.2 for ApoE ε2, ε3, and ε4, respectively) (Fig. 3b). There was no statistically significant difference between Hill coefficients of isoforms for murine TREM2 (*p* > 0.05).

**Fig. 4:**
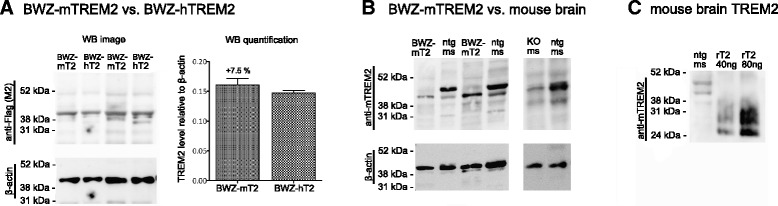
Rougly similar level of TREM2 in BWZ-mTREM2 and hTREM2 cells and lower than in mouse brain. **a** In both the BWZ-mTREM2 and BWZ-hTREM2 reporter cells, the TREM2 constructs harbor a FLAG-tag. A western blot with an antibody detecting the FLAG-tag expression in the reporter cells indirectly showed that the TREM2 level was slightly higher in the BWZ-mTREM2 than in the BWZ-hTREM2 cells, although this was not statistically significant (*n* = 2 for both cell lines). Protein loading was 30 μg (*lanes 1–2*) and 50 μg (*lanes 3–4*). **b** Western blot detection of murine TREM2 in BWZ-mTREM2 cells (“BWZ-mT2”) compared to a nontransgenic mouse brain (“ntg ms”). Protein loading was 30 μg (*lanes 1–2*) and 50 μg (*lanes 3–4*). TREM2 in the nontransgenic mouse brain was also compared to *Trem2* knockout mouse brain (“KO ms”). Protein loading was 40 μg in both *lanes*. **c** The level of TREM2 protein in the nontransgenic mouse brain was estimated to approximately 1 ng/μg protein by using recombinant mouse TREM2 (“recT2”) as a standard. Mouse brain extract (30 μg total protein) was loaded in *lane 1*, while 40 and 80 ng pure, recombinant TREM2 was loaded in *lanes 2* and *3*, respectively

In order to compare the expression levels of TREM2 in the BWZ-mTREM2 and BWZ-hTREM2 cells, western blots were semi-quantified with densitometry. We found that the expression level was 7.5% (±0.2%) higher in BWZ-mTREM2 than in BWZ-hTREM2 cells (Fig. 4a), although the difference was not statistically significant (*p* > 0.05; *n* = 2 for each cell line). We furthermore found that TREM2 protein expression in an SDS extract of the BWZ cells was lower than that of a brain SDS extract from a 14-month-old nontransgenic C57BL/6J mouse (≈1 ng TREM2/μg total protein; Fig. 4b, c). Only a faint co-migrating band was detected in the SDS-extract of *Trem2*-knockout mouse brain (Fig. 4b). The limitations of the western blot experiments are mentioned in the “Discussion” section.

### ApoE ε2, ApoE ε3, and ApoE ε4 bind to recombinant human TREM2 with high affinity

**Fig. 5:**
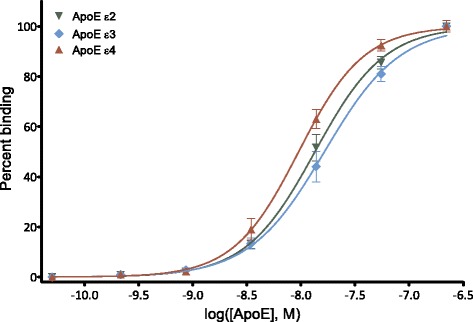
High-affinity binding of recombinant human ApoE ε2, ε3, and ε4 to recombinant human TREM2. In an ELISA-based binding assay with recombinant human TREM2 and recombinant human ApoE, ApoE ε2 (*inverted green triangle*), ApoE ε3 (*light blue diamond*), and ApoE ε4 (*red triangle*) isoforms bound to human TREM2 in vitro with *K*_d_ of 13, 16, and 9.5 nM, respectively. The points depict mean ± SEM of all responses that were measured in triplicates from three independent experiments. Data from each ApoE isoform was normalized individually as described in the “Methods” section. The *x*-axis shows the ApoE concentrations (in M) on a logarithmic scale

In order to determine affinity of the binding between ApoE and TREM2, we established an in vitro ELISA-based binding assay. Nonlipidated human ApoE bound to recombinant human TREM2 with high affinity. There were only small differences between isoforms with *K*_d_ of 13, 16, and 9.5 nM for ApoE ε2, ApoE ε3, and ApoE ε4, respectively (Fig. 5; ε2 −log*K*_d_ = 7.87 ± 0.02 M, ε3 −log*K*_d_ = 7.78 ± 0.03 M, ε4 −log*K*_d_ = 8.02 ± 0.03 M). The *K*_d_ of ApoE ε4 was lower than the *K*_d_ of ApoE ε3 (*p* < 0.001) and ApoE ε2 (*p* < 0.001). The Hill coefficients were similar for ApoE ε2 (1.4 ± 0.1), ApoE ε3 (1.3 ± 0.1), and ApoE ε4 (1.4 ± 0.1) (*p* > 0.05).

### Amino acids 130–149 in human ApoE contain a binding domain for human TREM2

**Fig. 6:**
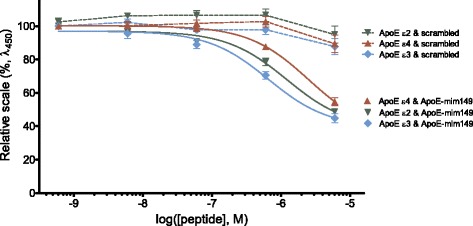
An ApoE-mimetic peptide (amino acids 130–149) reduced binding of human ApoE to human TREM2. An ApoE-mimetic (amino acids 130–149) or a scrambled peptide was allowed to compete with ApoE for binding to immobilized recombinant human TREM2 in the ELISA-based assay. This led to reduced binding of ApoE, isoforms ε2 (*inverted green triangle*), ε3 (*light blue diamond*), and ε4 (*red triangle*), to human TREM2 with *K*_i_ of 827, 475, and 1537 nM for ApoE ε2, ε3, and ε4, respectively. The points depict mean ± SEM of binding measured in duplicates from four independent experiments. The data was normalized to maximum (100%), which is binding of ApoE in the absence of a competing peptide, and minimum (0%), which is signal in the absence of ApoE as well as a TREM2-coat. The *x*-axis shows ApoE-mim149 or scrambled peptide concentration (in M) on a logarithmic scale

Previously, it has been found that the human ApoE fragment “ApoE-mim149” (amino acids 130-149 of ApoE) modulates microglial immune response [47]. To investigate whether this region contains a binding domain for human TREM2, we let ApoE-mim149 peptide and ApoE compete for binding to human TREM2. Adding human ApoE and ApoE-mim149 simultaneously to recombinant, human TREM2 resulted in reduced binding of ApoE to TREM2 with an increasing concentration of ApoE-mim149 in the mixture (*p* < 0.001 for all three ApoE isoforms at both 6 μM and 0.6 μM concentration of ApoE-mim149). This effect was not seen when instead an increasing concentration of a scrambled peptide (altered sequence of amino acids 130–149 of ApoE) was supplemented, indicating that a TREM2-binding region is present in amino acids 130–149 of human ApoE (Fig. 6). The *K*_i_ values for the three ApoE ε2, ε3, and ε4 with ApoE-mim149 present were 827, 475, and 1537 nM, respectively (ε2 −log*K*_i_ = 6.08 ± 0.1; ε3 −log*K*_i_ = 6.32 ± 0.1; ε4 −log*K*_i_ = 5.81 ± 0.1). The *K*_i_ for ApoE ε4 was significantly higher than for ApoE ε3 (*p* < 0.001).

## Discussion

*APOE* and *TREM2* are the two most predominant genetic risk factors in AD. Previous papers have reported binding between human ApoE and human TREM2 only in pure in vitro settings [34–36], but did not contain investigations on whether ApoE was an agonist mediating intracellular signaling through TREM2, or alternatively an antagonist inhibiting other stimuli. In this study, using cell reporter assays, we provide the first evidence that ApoE and TREM2 interact in a signaling pathway. With a sensitive ELISA-based binding assay, we have determined binding affinities of human ApoE ε2, ε3, and ε4 to human TREM2 to be in the low nM range. Importantly, we report the first evidence that a TREM2-binding domain is found in amino acids 130–149 of human ApoE, which is the conserved receptor-binding domain in the main ApoE isoforms, ε2, ε3, and ε4, as reviewed in [48].

By using an ELISA-based binding assay, we found a statistically significant lower *K*_d_ for ApoE ε4 (9.5 nM) than for ApoE ε2 and ApoE ε3 (13 and 16 nM, respectively), while the EC_50_ for human TREM2-signaling was similar among the ApoE isoforms (27, 33, and 34 nM for ApoE ε2, ε3, and ε4, respectively). Since we found a TREM2-binding domain in a conserved region of ApoE (amino acids 130–149), it is reasonable to expect equal binding affinity between the recombinant human ApoE isoforms, as was also previously reported [34, 35]. The biological relevance of potential isoform differences in TREM2 stimulation, if any, needs to be further investigated by in vivo modeling. The EC_50_ data found are in the physiologically relevant range, since the ApoE concentration is between 60–300 nM in the human cerebrospinal fluid (CSF) and 880–2700 nM in the plasma [49–53]. In the brain, it can generally be expected that the concentration of a given protein depends on location. The availability of a ligand to a receptor also depends on its binding to other proteins. Accordingly, ApoE is likely to be present in higher local concentrations in amyloid plaques and close to cell surfaces of astrocytes, and the CSF concentration gives only a rough estimate of the ApoE concentration in the brain. With western blots, we did not find evidence that TREM2 was overexpressed in the BWZ reporter cells as compared to the brain from a nontransgenic 14-month-old mouse. Using *Trem2* knockout brain tissue, we showed that the immunoreactive band in tissue extract of the nontransgenic mouse brain is largely derived from murine TREM2. The major limitation with the western blot experiments is that the binding of the TREM2 antibody might depend on TREM2 glycosylation, which presumably differs between native TREM2 in mouse brain and TREM2 in transfected BWZ cells, and surely with nonglycosylated recombinant TREM2. The TREM2 antibody used was chosen since it was raised against a domain (amino acids 154–165 of mouse TREM2), which is distinct from the location of the *N*-glycosylation, which occurs at amino acids 20 and 80 [54]. Yet, it still cannot be excluded that differential glycosylation still plays a role. A 40–45-kDa size of the TREM2 immunoreactive band from brain extract is quite consistent with previous observations [55]. Glycosylation structure and complexity affects migration of proteins on a polyacrylamide gel. The vector should encode a TREM2 fusion protein of ≈36 kDa, but since it is expressed in a cell line, the protein glycosylation is likely to be less complex than that of the native TREM2 protein in mouse brain.

By stimulating BWZ-hTREM2 reporter cells with ApoE, we found a significantly higher Hill coefficient for ApoE ε4 (1.7) than for ApoE ε2 (1.0). When using a sensitive ELISA-based binding assay, we found similar Hill coefficients between the ApoE isoforms when bound to recombinant, human TREM2. The relevance of binding differences needs to be further explored with other experimental techniques such as structural modeling of human TREM2 bound by ApoE isoforms.

It might be tempting to conclude that the risk of AD does not depend on ApoE/TREM2 signaling since we did not find evidence of ApoE isoform-dependent differences in binding to or signaling through TREM2. However, other factors could play a role in vivo: (1) It has been shown that the ApoE concentration in CSF depends on *APOE* genotype [51], making it plausible that individuals homozygous for *APOE4* exhibit diminished stimulation of TREM2 as a result of lower ApoE concentration; (2) the outcome of the ApoE-mediated intracellular signaling through TREM2 could vary depending on ApoE isoforms due to cofactors with different binding affinities exerting effects on structure and stability, or perhaps even downstream targets of the intracellular signaling through TREM2. Thus, it is still possible that the ApoE/TREM2 interaction contributes to the ApoE isoform-dependent AD risk.

Ligand-induced receptor signaling also depends on interactions with other constituents in the membrane and on anchoring proteins. Heparan sulfate proteoglycan (HSPG) is a possible cofactor that could be involved in the ApoE stimulation of TREM2. HSPGs are ubiquitously present on cell surfaces and in extracellular matrices, and the heparan sulfate (HS) side chains can bind ApoE [56–58]. Interestingly, both the receptor-binding region and the lipid-binding region of ApoE are involved in binding to HS [57–63]. HSPGs are known to regulate the interaction between ligands and their receptors [64–67]. In this sense, ApoE ε2 binds more readily to HS than ApoE ε3 [56, 68], thus perhaps differentiating the ApoE ε2-mediated activation of TREM2 from the ApoE ε3-mediated activation. Interestingly, HS and ApoE are both present in amyloid plaques in AD and animal models [69–73].

Another possible cofactor for the ApoE/TREM2 signaling is Aβ. While Aβ has been reported to bind both the lipid-binding region (amino acids 244–272) and the receptor-binding region (within amino acids 130–149) of ApoE [26, 74], it is interesting to note that Aβ binding to the lipid-binding region of ApoE would allow for the possibility of stimulation of TREM2 by the Aβ/ApoE complexes found in or around amyloid plaques.

A link between ApoE and Aβ has been found consistently, as *APOE* dose-dependently enhances the risk of developing late-onset AD and also increases amyloid burden among those carrying *ε4* alleles [15, 75]. Likewise, in APP^V717F^ transgenic mice, the ApoE ε4 isoform was found to increase Aβ deposition [28] and decrease Aβ clearance [32], although the specific clearance pathway involved is still incompletely understood. In C57BL/6 mice, ApoE (lipid-poor or lipid-rich) and Aβ (monomeric Aβ_40_ or Aβ_42_) were injected intracerebrally either alone or in ApoE-Aβ complexes. The authors found decreased Aβ clearance associated with the ApoE ε4 isoform [76]. Since ApoE is associated with Aβ in amyloid plaques and TREM2 is found in microglia or macrophages adjacent to the plaques [6, 7], it is intriguing to think that there might be a connection between Aβ/ApoE complexes and TREM2 on surrounding microglia/macrophages.

While reduced ApoE gene dosage consistently ameliorates AD phenotypes [30, 33], there has been conflicting reports on gene dosage effects of TREM2 in AβPP transgenic mice [7, 77, 78]. Reduced TREM2 gene dosage was found to enhance amyloid pathology in one of the studies [78], while it decreased amyloid pathology in another study [7]. Importantly, the latter group recently showed that TREM2 deficiency in APP/PS1 mice lead to reduced amyloid burden at an early disease stage, while it enhanced amyloid burden at a late disease stage [79]. When AD pathogenesis is investigated in transgenic mouse models, human ApoE isoforms are often expressed, either by using a heterologous promoter [80] or by gene replacement in animals devoid of murine ApoE [81]. Knowing that the TREM2/ApoE interaction is presumably relevant to AD pathogenesis and perhaps also to other neurodegenerative diseases, we found it essential to determine the extent to which human ApoE stimulated murine TREM2 signaling. Such information is needed to better interpret studies with mouse models of disease. We found that all three human ApoE isoforms were agonists to murine TREM2 albeit with slightly reduced efficacy compared to human TREM2. Therefore, double transgenic ApoE/AβPP animal models might inadequately reflect the interactions between ApoE and TREM2 in the AD brain; microglial activation triggered by ApoE stimulation of murine TREM2 might be more modest in transgenic mice than when TREM2 in AD brain is exposed to the same stimuli. Thus, it could be worth the effort to create human *TREM2* knock-in models, or at least to insert exons encoding the extracellular domains of human *TREM2* into the murine genome.

The ApoE/TREM2 interaction as observed by us might be involved in a clearance pathway, which is consistent with experiments with transgenic mice convincingly linking ApoE to Aβ clearance [28, 32]. ApoE could potentially also regulate responses of macrophages or microglia to specific stimuli of neuroinflammation and lipid metabolism and activate specific cellular functions such as phagocytosis. Microglia do not express TREM2 constitutively on the cell surface [82], thus complicating investigations of ligand-receptor interactions with primary cells in vitro. The reporter cells used here serve as an efficient and sensitive tool delivering reliable quantitative data on ligand-receptor interactions and signaling. However, there might be differences in the cell-surface expression of proteins or glycoproteins (possibly functioning as cofactors to TREM2) as well as intracellular signaling cascade between reporter cells and microglia/macrophages.

In the plasma, the ApoE isoforms differentially bind to lipids, with ApoE ε2 and ε3 preferring high-density lipoprotein (HDL) lipids and ApoE ε4 preferring low-density lipoproteins (LDL) and very low-density lipoprotein (VLDL) lipids [48, 83–86]. However, the intact BBB of the adult brain confers a barrier through which neither lipoproteins nor lipids can pass, thus creating a restrictive environment that is distinct from the plasma. In this manner, LDL and VLDL are not present in the CNS, and the astroglial-derived HDL particles are structurally different from those found in the plasma (see [18] for a review on CNS lipoproteins). However, the lipoprotein-affecting enzymes, transporters, and receptors present in the periphery are also found in the CNS [18], and different binding affinities have been reported for the ApoE isoforms to their receptors, which can also be affected by ApoE lipidation state for some receptors [76, 87, 88]. Using reporter cells, it was recently found that various anionic lipids found in AD amyloid plaques were agonists for TREM2 [78]. Since ApoE is a lipid carrier, the ApoE/TREM2 interaction is likely to be involved in lipid sensing by TREM2 [78, 89], although we hereby demonstrate that the nonlipidated ApoE protein is sufficient for a high-affinity interaction. Initial studies of TREM2 ligand binding suggested that TREM2 could function as a pattern recognition receptor distinguishing negatively charged motifs [37]. In addition to ApoE binding, other apolipoproteins, ApoA1, ApoA2, ApoB, and ApoJ, also bind to TREM2 [34–36]. Binding of lipidated as well as nonlipidated human ApoE to human TREM2 has been demonstrated [34–36]. In our cell reporter assays, ApoE is bound to a Maxisorp plate, and it is unlikely that lipids from cells or FBS will lipidate ApoE under these circumstances. Also arguing against the necessity of ApoE being lipidated in order to bind TREM2 is our demonstration of high affinity binding between recombinant ApoE and recombinant TREM2 in a cell-free and lipid-free ELISA binding assay. Moreover, the dose-response curves for in vitro binding data and reporter cell signaling data were quite similar, which further argues against lipidation of ApoE being necessary for TREM2 signaling. Finally, the ApoE fragment, ApoE-mim149, binds to TREM2. This peptide is derived from the receptor-binding domain of ApoE, which is distinct from the lipid-binding domain [48]. It is conceivable that immobilization of ApoE to a plate can affect its conformation such that an ApoE conformer is created, which is prone to stimulate TREM2 in the reporter assay. However, ApoE interacting with TREM2 and displaying EC_50_ and *K*_d_ values in the same approximate concentration range when immobilized (reporter assay) and when in solution (binding and competition assays) argues that such a potential effect is either very limited or not relevant in our experiments.

## Conclusions

In conclusion, we have proved the existence of a joint signaling pathway between TREM2 and ApoE, the proteins encoded by the two strongest genetic risk factors for AD, and found that the human ApoE isoforms ε2, ε3, and ε4 are all agonists to human and murine TREM2, albeit with reduced potency to murine TREM2. We have also determined binding affinities of the human ApoE isoforms to human TREM2 and identified a potential TREM2-binding domain in the receptor-binding region of human ApoE, which is conserved among the human isoforms. Our findings lay base for further studies on the pathophysiological relevance of the ApoE-dependent TREM2 receptor signaling. Within preclinical pharmacology, our work points out the need to develop new transgenic models with human TREM2 expression as to properly display the interplay between ApoE and TREM2 on AD phenotypes and to evaluate therapeutic strategies.

## Additional file

Additional file 1: Supplementary Material. (DOCX 1372 kb)

## Abbreviations

AD: Alzheimer’s disease
ApoE: Apolipoprotein E
Aβ: Amyloid-beta
AβPP: Amyloid-beta precursor protein
BSA: Bovine serum albumin
CPRG: Chlorophenol-red β-*D*-galactopyranoside
CSF: Cerebrospinal fluid
DAP12: DNAX activating protein of 12 kDa
DTT: Electran 1,4-dithiothreithol
EDTA: Ethylenediaminetetraacetic acid
FBS: Fetal bovine serum
HDL: High-density lipoprotein
HRP: Horseradish peroxidase
HS: Heparan sulfate
HSPG: Heparan sulfate proteoglycan
ITAM: Immunoreceptor tyrosine-based activation motif
LDL: Low-density lipoprotein
LDLR: Low-density lipoprotein receptor
NFAT: Nuclear factor of activated T cells
PBS: Phosphate-buffered saline
PMA: Phorbol myristate acetate
RPMI: Roswell Park Memorial Institute 1640 medium
RT: Room temperature
SDS: Sodium dodecyl sulfate
TBS: Tris-buffered saline
TIM2: T cell immunoglobulin domain and mucin domain 2
TREM2: Triggering receptor expressed on myeloid cells 2
VLDL: Very low-density lipoprotein
ZAP-70: Zeta-chain associated protein kinase 70
