# Supplementary material for: The Alzheimer’s disease risk factors apolipoprotein E and TREM2 are linked in a receptor signaling pathway
Source: J Neuroinflammation. 2017 Mar 21;14:59. doi: 10.1186/s12974-017-0835-4 (PMC5360024; doi:10.1186/s12974-017-0835-4)
Supplement: Supplementary file 1 — Supplementary Material. (DOCX 1372 kb) [file 12974_2017_835_MOESM1_ESM.docx]

**Supplementary Material**

**Supplementary Figures:**


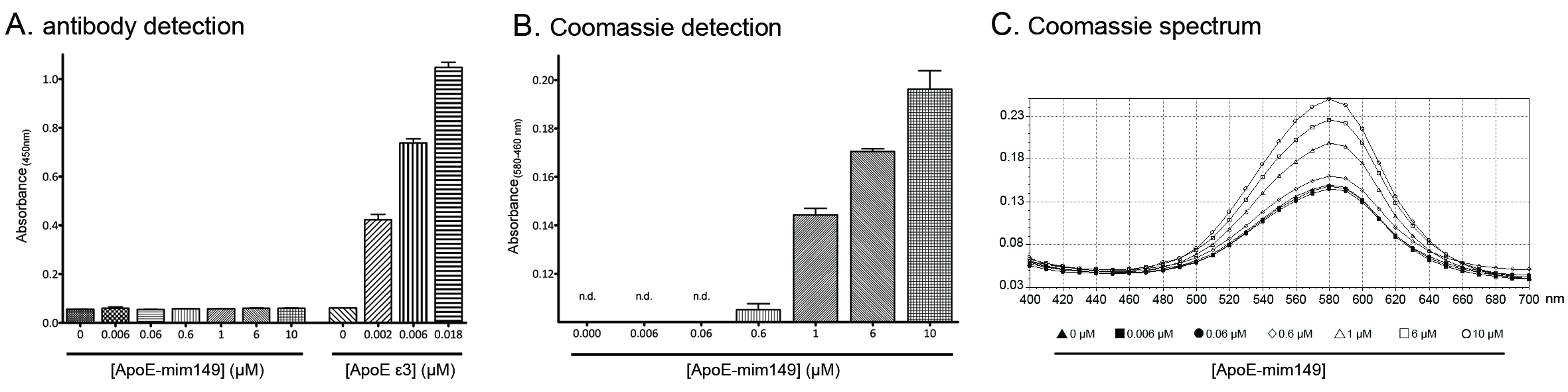


Figure S1: ApoE-mim149 is not recognized by anti-ApoE antibody #178479.

A) ApoE-mim149 (0.006-10 µM) and ApoE (0.002-0.018 µM) were coated to a Maxisorp plate. Polyclonal anti-ApoE antibody (#178479) bound to ApoE, but not to ApoE-mim149, even at a high concentration (10 µM) of ApoE-mim149. Columns represent mean ± S.E.M. from two independent experiments with duplicates. B) Coomassie Brilliant Blue was used to verify that ApoE-mim149 was bound to the Maxisorp plate at the given concentrations. Background absorbance at 460 nm was subtracted from the maximum absorbance at 580 nm. Columns represent mean ± S.E.M. from two independent experiments with duplicates. n.d.: not detectable. C) A representative spectral analysis of the Coomassie Brilliant Blue stain of ApoE-mim149 when bound to the Maxisorp plate. The maximum absorbance was found at 580 nm.

**Supplementary methods:**

**ApoE3 and ApoE-mimetics149 detection assay (Figure S1A)**

Transparent Maxisorp 96-well plates were coated with ApoE ε3 and ApoE-mim149 at various concentrations for 90 min at 37 °C in PBS. The plates were blocked with 1 % (w/v) BSA in PBS for 1 hour at 37 °C before being incubated with goat anti-ApoE antibody (18 ng/mL) followed by 30 min at RT with anti-goat-HRP antibody (0.03 µg/mL). Finally, the assays were developed with K-blue aqueous substrate (TMB) and absorbance was measured at 450 nm with a SpectraMax 190 plate reader (Molecular Devices).

**Detection of ApoE-mim149 coating (Figure S1B-C)**

Transparent Maxisorp 96-well plates were coated with ApoE-mim149 at various concentrations for 90 min at 37 °C in PBS (pH7.4) followed by washing with PBS and dH_2_O. Plates were then incubated with Coomassie Brilliant Blue (#1610400; Bio-Rad) diluted 0.1 % in dH_2_O with 50 % (v/v) methanol (#20847.320; VWR) and 10 % (v/v) glacial acetic acid (#RH1019; Rathburn, Walkerburn, Scotland) for 15 min at RT followed by washing with dH_2_O. 0.075 % SDS in PBS was added to each well, incubated for 5 min at RT and measured at 460 nm and 580 nm with a SpectraMax 190 plate reader (Molecular Devices).
